# Pnmt-Derived Cardiomyocytes: Anatomical Localization, Function and Future Perspectives

**DOI:** 10.3389/fphys.2019.00713

**Published:** 2019-07-10

**Authors:** Xuehui Fan, Tianyi Sun, William Crawford, Xiaoqiu Tan, Xianhong Ou, Derek A. Terrar, Steven N. Ebert, Ming Lei

**Affiliations:** ^1^ Key Laboratory of Medical Electrophysiology, Ministry of Education and Medical Electrophysiological Key Laboratory of Sichuan Province, Collaborative Innovation Center for Prevention of Cardiovascular Diseases, Institute of Cardiovascular Research, Southwest Medical University, Luzhou, China; ^2^ Department of Pharmacology, University of Oxford, Oxford, United Kingdom; ^3^ Burnett School of Biomedical Sciences, College of Medicine, University of Central Florida, Orlando, FL, United States

**Keywords:** optogenetics, Pnmt-derived cardiomyocytes, heart, Pnmt, catecholamines

## Abstract

In this mini-review, we provide an overview of phenylethanolamine-N-methyl transferase (Pnmt)-derived cardiomyocytes (PdCMs), a recently discovered cardiomyocyte subpopulation. We discuss their anatomical localization, physiological characteristics, possible function, and future perspectives. Their unique distribution in the heart, electrical activity, Ca^2+^ transient properties, and potential role in localized adrenergic signaling are discussed.

## Introduction

The advent of cardiac optogenetics has facilitated the identification of a new cardiomyocyte subpopulation in the murine heart, phenylethanolamine-N-methyl transferase (Pnmt)-derived cardiomyocytes (PdCMs) (Wang et al., 2017). Through selective manipulation of these cells using optogenetics, our study demonstrated how this technique can be used to target specific cardiac cellular subpopulations, overcoming many of the issues associated with traditional electrophysiological techniques. In addition, combining optogenetics with electrophysiology, voltage imaging, and Ca^2+^ imaging allows for elucidation of specific cell functions *in vivo*. In this mini-review, we will discuss the anatomical localization, physiological characteristics, possible function, and future perspectives of PdCMs.

## Early Studies of Catecholamines in the Heart

Catecholamines are endogenous signaling molecules such as dopamine, noradrenaline (norepinephrine), and adrenaline (epinephrine). These molecules are typically synthesized in neurons at the site of their release, both at neuronal terminals and in cell bodies. The detection of catecholamines in the heart dates back to the late 1960s by Ignarro and Shideman, who demonstrated their production by cells other than sympathetic neurons (Ignarro and Shideman, 1968a,b,c). They determined the time of appearance and concentration of dopa (DA), dopamine, norepinephrine, and epinephrine in developing chick hearts, demonstrating the presence of catecholamines during the early days of the development. Embryonic hearts were also noted to take up tritium-labeled norepinephrine (H^5^-NE) and epinephrine (H^3^-E) during the development (Ignarro and Shideman, 1968b). The presence of catecholamine biosynthetic enzymes, including tyrosine hydroxylase, dopa decarboxylase, dopamine β-oxidase, and Pnmt were also confirmed in chick embryonic hearts (Ignarro and Shideman, 1968c). Furthermore, catecholamine metabolism enzymes including catechol-O-methyl transferase (COMT) and monoamine oxidase (MAO) expression were also detected in the embryonic and developing chick heart. These enzymes were first detected in the embryonic developing heart on the fourth day, and increased thereafter until the 10th day when their activities plateaued. Activities of COMT and MAO increased on the 15th and 16th days and attained maximal values by the 19th day. Both enzymes subsequently exhibited sharp declines in their activities (to 13 and 40% of maximal values, respectively), reaching minimal values on the second and third days after hatching. This study therefore demonstrated the presence of catecholamines, their biosynthetic enzymes, and catecholamine transporters in the heart, although it did not determine their cellular origin.

## Adrenergic Cells and Intrinsic Cardiac Adrenergic Cells

Subsequent studies from numerous groups have established the existence of adrenergic cells in the heart (Ziegler et al., 2002; Owji et al., 2013). Adrenergic cells are present in both peripheral tissues (e.g., the adrenal medulla) and the central nervous system (e.g., the ventrolateral medulla) (Ziegler et al., 2002; Owji et al., 2013). Their primary role is mediating sympathetic responses to potentially stressful stimuli. These cells are characterized by their expression of Pnmt, the enzyme responsible for the final step in the adrenergic biosynthetic pathway, converting norepinephrine to epinephrine. The expression level of Pnmt in adrenergic cells within adrenal medulla is much higher than those in the brain and other tissues (Ziegler et al., 2002; Owji et al., 2013). Intriguingly, similar adrenergic cells have been identified in the heart of both developing and mature adults, and have been termed “Intrinsic Cardiac Adrenergic” (ICA) cells (Ebert et al., 1996, 2004; Huang et al., 1996, 2005; Ebert and Thompson, 2001; Osuala et al., 2011). ICA cells are present across all four chambers of the developing heart, but are primarily concentrated on the left side of the heart by adulthood, located predominantly in the left atrium and specific regions of the left ventricle (Owji et al., 2013). Such early expression of *Pnmt* is believed to denote a mesodermal origin of adrenergic cells in the heart (Ebert et al., 1996; Huang et al., 1996). Somewhat later in development, neural crest-derived (NCD) cells invade the heart and also appear to contribute neuroendocrine functions (Owji et al., 2013). These cells contain *Pnmt* mRNA and produce epinephrine (Ebert et al., 1996; Huang et al., 1996). *In vitro* experiments demonstrate the ability of these cells to increase the contraction rates of neonatal rat cardiomyocytes in culture. These Pnmt-positive cells have also been identified in human fetal hearts before sympathetic innervation of the heart occurs (Huang et al., 1996). However, the physiological role of these neuroendocrine cells within the heart is still poorly understood.

## Optogenetic Illumination of Pnmt-Derived Cardiomyocytes

We recently developed a novel optogenetic mouse model with conditional cell-type expression of ChR2 by using *Pnmt* as a prompter gene (Wang et al., 2017). In this study, we crossed Pnmt-Cre mice with Ai27D mice (Stock No. 012567, Jackson Labs) expressing an improved channelrhodopsin-2/tdTomato fusion protein. Following exposure to Cre recombinase in PdCMs, the offspring mice were used in optogenetic studies with rapid depolarization of PdCMs by illumination with blue light (450–490 nm). This allowed us to study their physiological properties in intact cardiac tissue (Wang et al., 2017). The original aim of generating such a model was to create a tool to study adrenergic cells and adrenergic-derived cells selectively. We subsequently identified a distinct subpopulation of Pnmt-derived cardiomyocytes in murine heart (Wang et al., 2017). The genotypes of offspring showed successful cell-type specific expression of Channelrhodopsin-2 (ChR2)-tdTomato under control of *Pnmt* promoter in Pnmt-Cre-ChR2 animals. Several types of ChR2/tdTomato fluorescence positive cells were identified based on their morphology: small triangular-shaped cells with positive Pnmt immunofluoresent staining that primarily appeared to be located in interstitial spaces. These cells are consistent with the ICA cells previously reported in the literature. A large number of elongated, rod-shaped ChR2/tdTomato positive cells were also found. Based on their myocyte appearance, and the expression of α-actinin (myocyte marker), we confirmed that they are cardiomyocytes, and termed them Pnmt -derived cardiomyocytes (PdCMs) (Wang et al., 2017).

## Unique Distribution of Pnmt-Derived Cardiomyocytes

**Figure 1:**
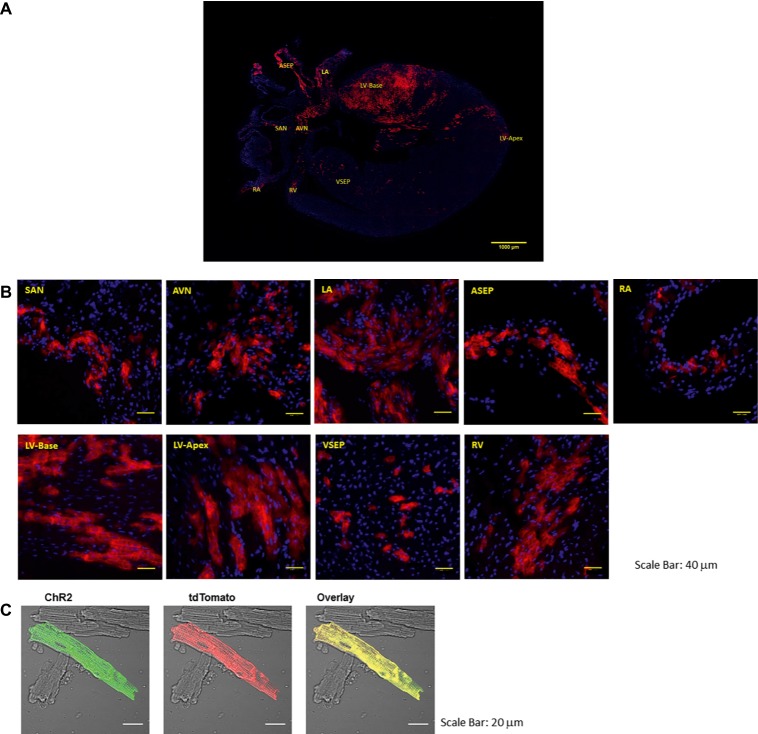
Representative images of the coronal section and selected regions from the section of an adult ChR2/tdTomato mouse heart showing fluorescence and morphology of the ChR2/tdTomato positive cells. **(A)** A representative coronal section from an adult ChR2/tdTomato mouse heart; **(B)** inserts of zoom-in views showing tdTomato fluorescence in different regions of the heart; the labeling of the inserts indicates the corresponding locations as marked in **(A)**. SAN, sinoatrial node; AVN, atrioventricular node; LA, left atrium; RA, right atrium; LV, left ventricle; RV, right ventricle; ASEP, atrial septum; VSEP, ventricular septum. **(C)** Immunostaining of ChR2 with anti-ChR2 antibody; tdTomato fluorescence in isolated LV cardiomyocytes; overlay showing co-localization of ChR2 and tdTomato [Adapted with permission from Wang et al. (2017) and Ni et al. (2017)].

PdCMs have a unique distribution to the left heart and cardiac conduction system (Ni et al., 2017; Wang et al., 2017). As shown in Figure 1, tdTomato fluorescence in coronal heart sections taken from Pnmt-Cre-ChR2 mice reveals a strong lateralization to the left side of the heart, dispersed throughout the left atrium and left ventricle. Concentrated ChR2-tdTomato fluorescence was also observed throughout the cardiac conduction system, including the sinoatrial node and atrioventricular node. Pnmt-Cre-ChR2 heart sections were stained with the myocyte marker α-actinin to demonstrate co-localization of α-actinin and tdTomato in the left ventricle, providing compelling evidence that many Pnmt-derived cells are cardiomyocytes. SAN and AVN sections were also stained for the pacemaker cell marker HCN4, and demonstrated co-localization with tdTomato. Interestingly, areas with high PdCMs density overlap with the area of rich sympathetic innervation (Kimura et al., 2012). It is not yet known whether this corresponding distribution suggests some kind of functional connection between Pnmt-derived cardiomyocytes with sympathetic nerve endings. Future investigation into this relationship may reveal important localized cardiac regulation mechanisms.

## Electrophysiological Properties of Pnmt-Derived Cardiomyocytes

The excitability and contractility of PdCMs has been studied using optogenetic models (Ni et al., 2017; Wang et al., 2017). Whole *ex vivo* Pnmt-Cre-ChR2 hearts were isolated and stimulated using 470-nm light pulses. We observed a clear electrical response correlated to the distribution of PdCMs. Targeted light pulses were shown to trigger excitation of the left atrium and left ventricle, but not of the right atrium and right ventricle. The light-based excitability of Pnmt-derived cardiomyocytes was also demonstrated using optical mapping of voltage and Ca^2+^ signals, in dissected atrial tissue or *ex vivo* hearts in Pnmt-Cre-ChR2 mice. This confirmed equivalent optogenetic triggering abilities of atrial and ventricular membrane voltage and intracellular Ca^2+^ signals (Ni et al., 2017; Wang et al., 2017). The contractile ability of PdCMs was also confirmed in individual isolated PdCMs (Ni et al., 2017; Wang et al., 2017). Direct optogenetic stimulation triggered contraction of PdCMs, but not of conventional myocytes isolated from Pnmt-Cre-ChR2 mouse heart, confirming intact excitation-contraction coupling in Pnmt-derived cardiomyocytes (Ni et al., 2017; Wang et al., 2017). This demonstrates the mechanical response triggered by light-sensitive channels (ChR2s) and illustrates the feasibility for direct optogenetic control in cardiomyocyte contraction at single cell level.

## A Potential Role in Localized Adrenergic Signaling

Although a subset of Pnmt-derived cardiomyocytes express Pnmt, they do not produce tyrosine hydroxylase or dopamine β-hydroxylase, and therefore cannot synthesize norepinephrine. Most of the norepinephrine released from cardiac sympathetic nerve endings is reabsorbed into the nerve endings through the peripheral norepinephrine transporter (uptake-1 mechanism), and is recycled or metabolized in the cytoplasm of neurons (Ziegler et al., 2002). In addition to the neuronal uptake-1 mechanism, norepinephrine can also be taken up by nonneuronal tissues, including cardiomyocytes, *via* the uptake-2 mechanisms (Ziegler et al., 2002). The NA uptake-2 mechanism is facilitated by transporter protein 3 (EMT/OCT3) (Ziegler et al., 2002). As some Pnmt-derived cardiomyocytes express Pnmt, they may possess the ability to uptake norepinephrine through uptake-2 mechanism and subsequently convert it to epinephrine. Such potential role of Pnmt-derived cardiomyocytes needs to be further examined.

It is known that autonomic dysfunction including sympathetic hyperactivity often coincides with cardiac pathologies including heart failure, diabetic cardiomyopathy, myocardial ischemia, and infarction (Ma et al., 1997; Ishise et al., 1998; Motte et al., 2005). It is an important cause of ventricular arrhythmia, and is therefore a predictive index of morbidity and mortality (La Rovere et al., 1998; Nolan et al., 1998). Catecholamines and their oxidation products cause a direct toxic effect on the myocardium. Catecholamine-mediated myocardial stunning has been implicated in the pathogenesis of stress-induced cardiomyopathy in both animal models and human subjects. (Ueyama et al., 2002; Kassim et al., 2008; Gupta et al., 2018)

Biopsy of the myocardium in patients with pheochromocytoma or stress-induced cardiomyopathy shows similar histological findings. The clinical features in pheochromocytoma-related cardiomyopathy include hypertension, dilated or hypertrophic cardiomyopathy, and pulmonary edema due to cardiogenic and noncardiogenic factors, cardiac arrhythmias, and even cardiac arrest. Stress-related cardiomyopathy such as takotsubo cardiomyopathy occurs primarily in postmenopausal women. It has been suggested that lack of estrogen replacement in the postmenopausal state may predispose women to takotsubo cardiomyopathy (Kuo et al., 2010; Gupta et al., 2018).

## Open Questions and Future Perspectives

### How Pnmt-Derived Cardiomyocytes Are Thought to Arise in Heart Development?

**Figure 2:**
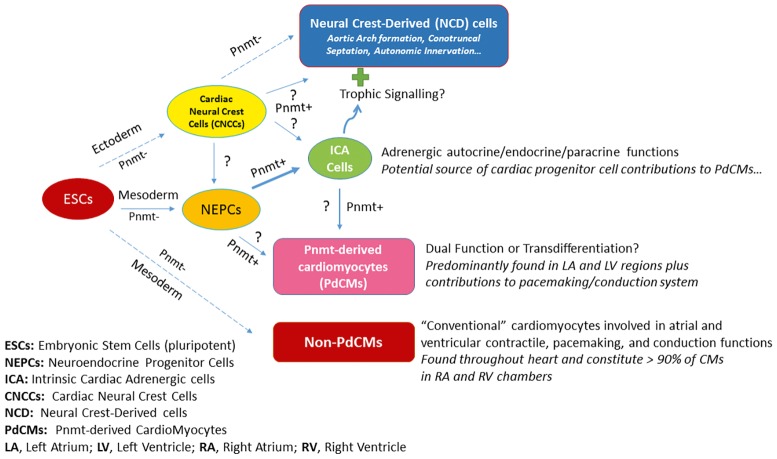
Schematic depiction of how PdCMs are thought to arise in heart development.

Figure 2 illustrates the schematic depiction of how PdCMs are thought to arise in heart development. By the blastocyst stage of embryonic development, pluripotent embryonic stem cells (ESCs) form and give rise to the ectoderm, mesoderm, and endoderm layers and cell types. A portion of mesoderm progenitors become committed to heart development (i.e., cardiac progenitor cells, CPCs – not shown), some of which become neuroendocrine progenitor cells (NEPCs) in the heart. A subset of these cells express Pnmt (Pnmt^+^ cells) and become intrinsic cardiac adrenergic (ICA) cells (Ebert et al., 1996; Huang et al., 1996) that appear in the heart prior to cardiac neural crest cell (CNCC) invasion (Ebert and Thompson, 2001). Fate-mapping studies with Pnmt-Cre/LacZ and Pnmt-Cre/ChR2 have shown that some of these ICA cells appear to transdifferentiate further into PdCMs (Ebert et al., 2004; Osuala et al., 2011; Ni et al., 2017; Wang et al., 2017). Notably, most PdCMs studied to date do not appear to actively express Pnmt, though some appear to retain that capability or may transiently express markers for both ICA and myocyte phenotypes for some duration during the transdifferentiation process (Owji et al., 2013). There are a number of open questions regarding this model as illustrated by the question marks (?) in Figure 2. It is unclear, for example, if neural crest cells also contribute to NEPCs and ICA cells in the heart. There are clearly Pnmt^+^ NCD cells in the vicinity of the heart, but studies have yet to demonstrate any specific contributions made by NCD Pnmt^+^ cells in heart development, though there is certainly evidence that ICA cells are located in the myocardial layers of the conotruncal regions, and it is well known that signals emanating from these regions are important for epithelial-mesenchymal transformations leading to septation. Further, there is strong evidence that the aortic arches are exquisitely sensitive to epinephrine during their formative period as topical administration of epinephrine leads to increases in aortic arch anomalies in the chick embryo model (Hodach et al., 1974, 1975). Given the spatiotemporal proximity of ICA cells during these formations, we speculate that they may exert trophic (paracrine) actions to influence the development of NCD cells/tissues including aortic arches, conotruncal septation, and/or autonomic innervation of the heart. While most cardiomyocytes in the heart are not derived from Pnmt^+^ cells (non-PdCMs), there are surprisingly large proportions of the LV and LA as well as the pacemaking and conduction cardiomyocytes that are marked by Pnmt expression at some point in their history (PdCMs). The most likely source of these PdCMs is ICA cells, but we cannot yet rule out that there could be other progenitor cell types such as NEPCs, non-PdCMs, or other as yet unidentified cell types that may also contribute to their production. Once cells become PdCMs, most of them appear to lose the ability to express Pnmt and other neuroendocrine markers, though some may retain for a period of time and may thus serve “dual function” roles in that they have specialized cardiomyocyte phenotypes (e.g., atrial and ventricular contractile, pacemaking, conduction) in addition to NEPC or ICA-like phenotypes.

### The Physiological Role of Phenylethanolamine-N-Methyl Transferase-Derived Cardiomyocytes

The physiological role of PdCMs requires further investigation. There are many unanswered questions, such as whether PdCMs have any adrenergic endocrine or paracrine role. It seems that they do not for the most part, though we do not have quantitative evidence for this. Some do indeed express Pnmt, but to be adrenergic, they would also need to express the three other enzymes in the pathway or at the very least be able to take up norepinephrine, which could then be converted to epinephrine by Pnmt. They would also have to possess secretory capability into adulthood as Huang et al. showed is present in early development (Huang et al., 1996). From our own studies, we have generally seen an inverse relationship between adrenergic endocrine and myocyte phenotypes, though some overlap has been observed. In other words, the question is whether PdCMs arise from ICA cells that gain a myocyte phenotype, do they transdifferentiate from ICA cells into myocytes, or some of both? Furthermore, if PdCMs are not adrenergic (i.e., arise from transdifferentiation), can they become adrenergic again (reverse transdifferentiate back to earlier phenotype)? If so, under what conditions might that occur? In conclusion, our understanding of biology and physiological function of PdCMs is limited, future efforts with a focus on the above challenging issues would greatly improve such understanding.

## Author Contributions

TS, XF, SE, and ML contributed to the draft-writing. WC, XT, XO, and DT contributed to the editing and proof-reading. SE and ML produced the Figures. ML is responsible for the correction, editing, and final approval.

### Conflict of Interest Statement

The authors declare that the research was conducted in the absence of any commercial or financial relationships that could be construed as a potential conflict of interest.
